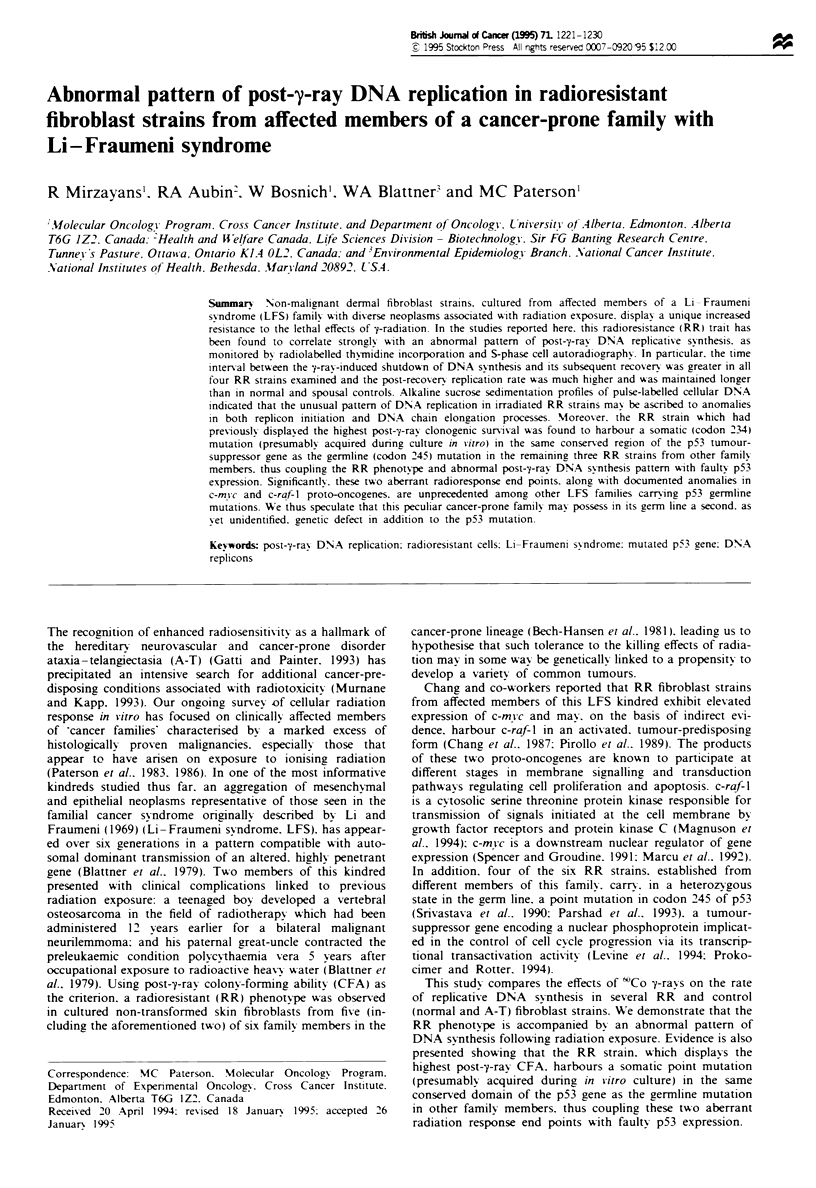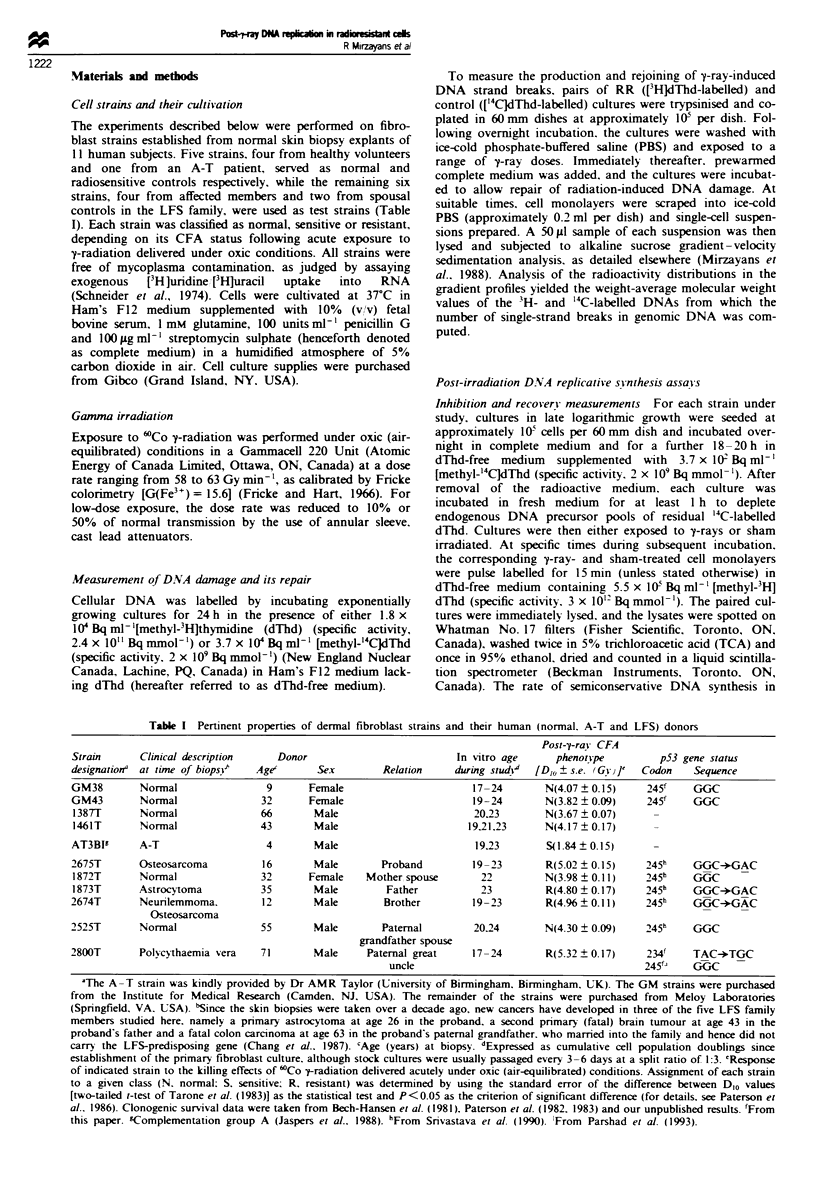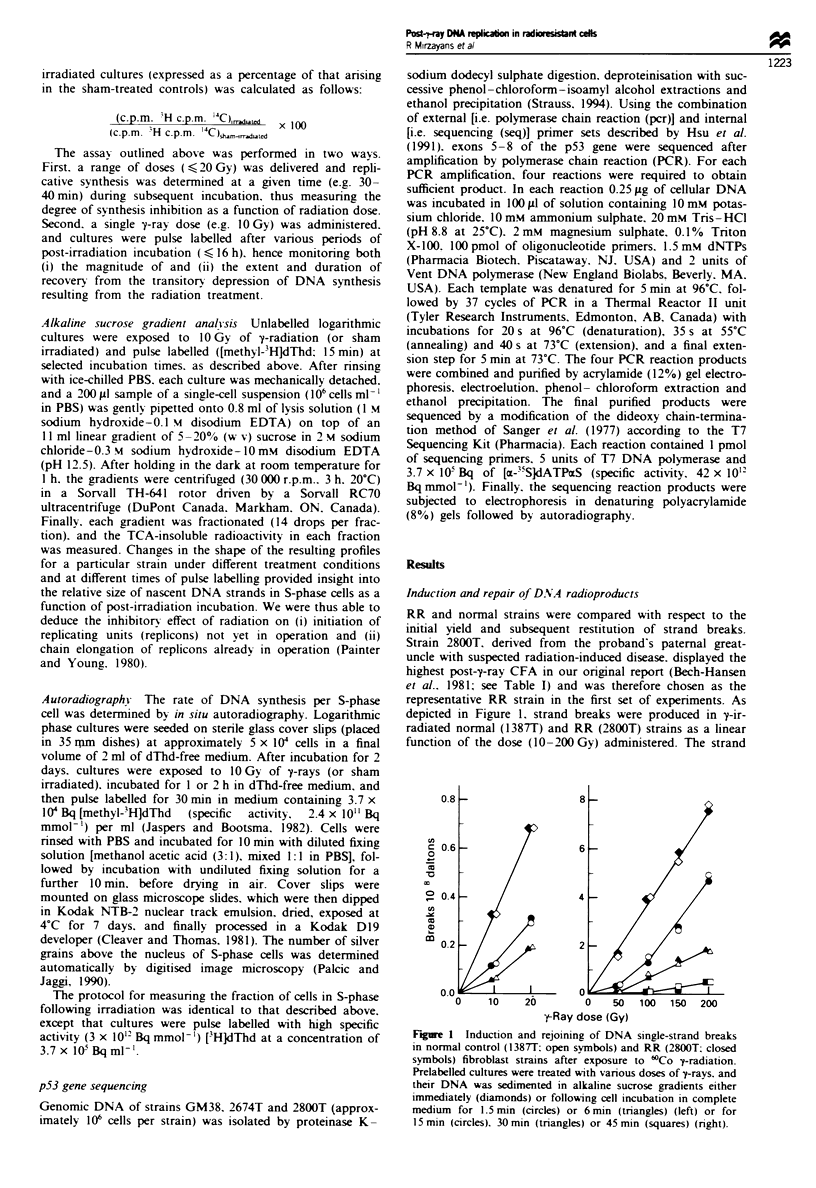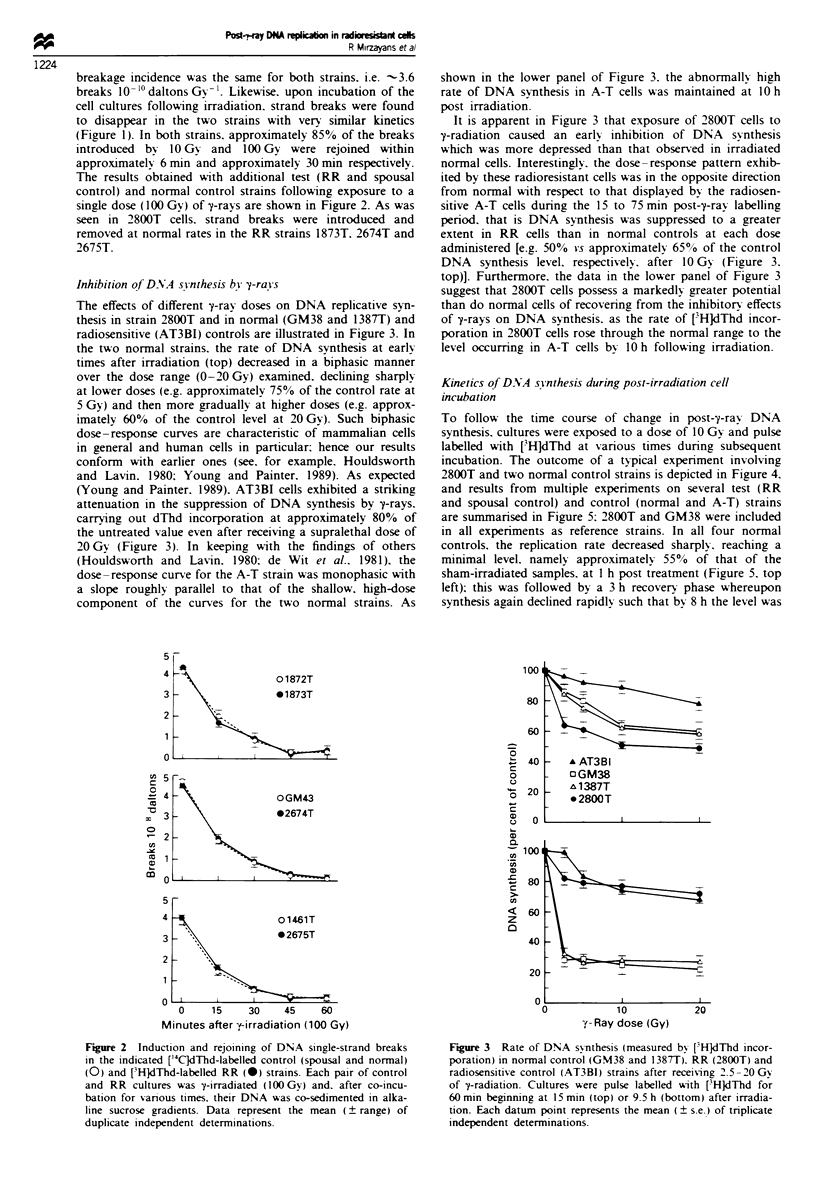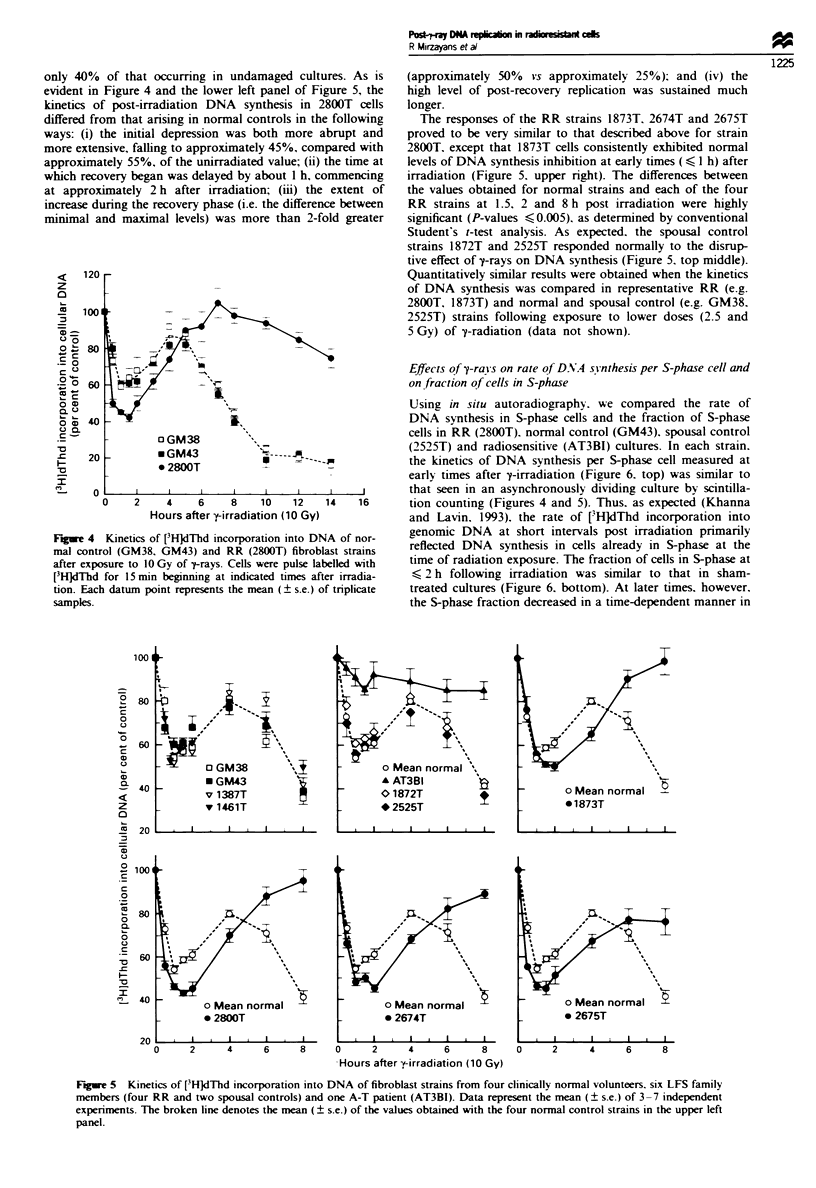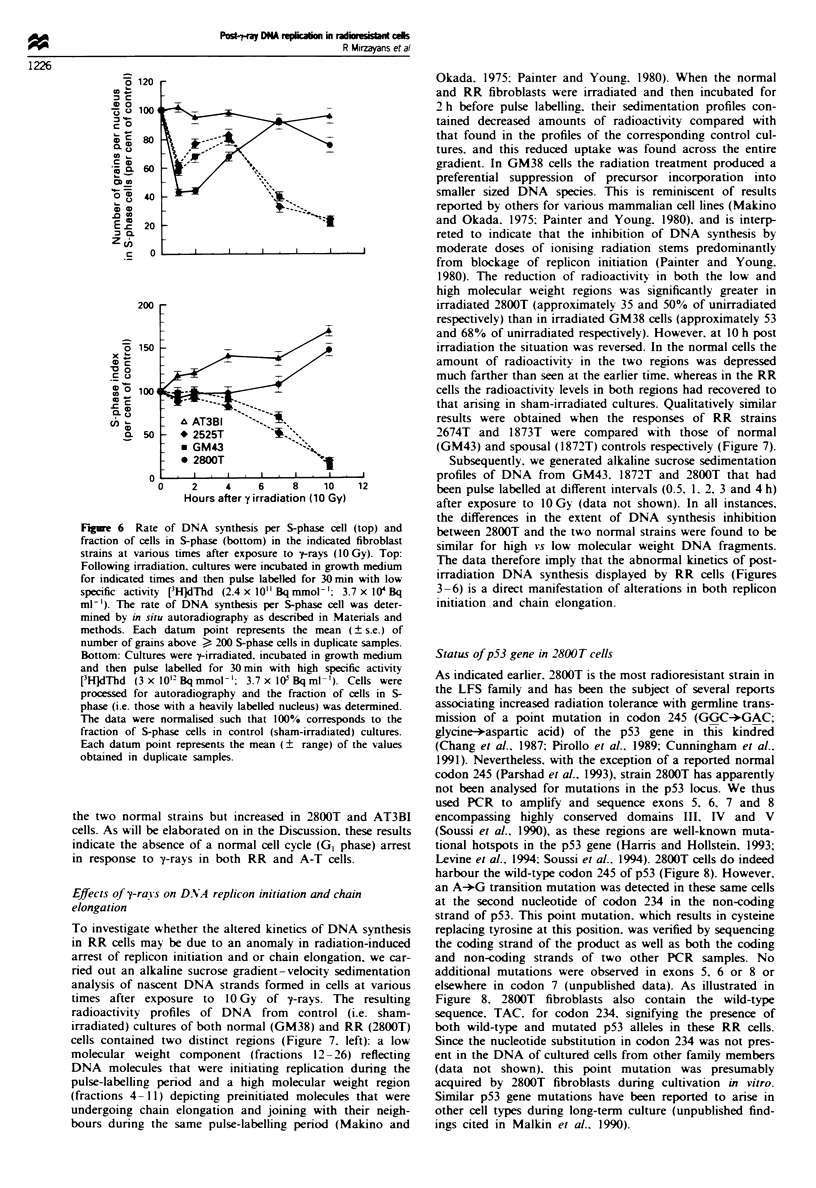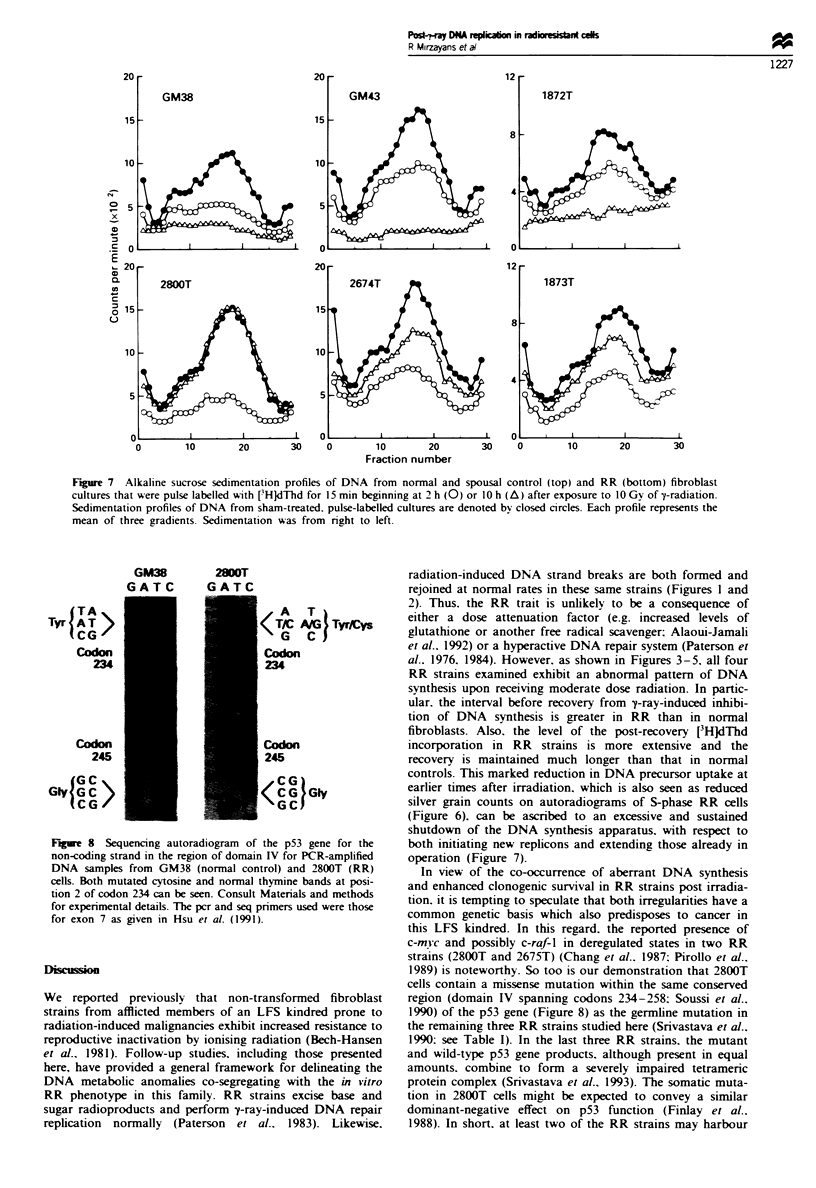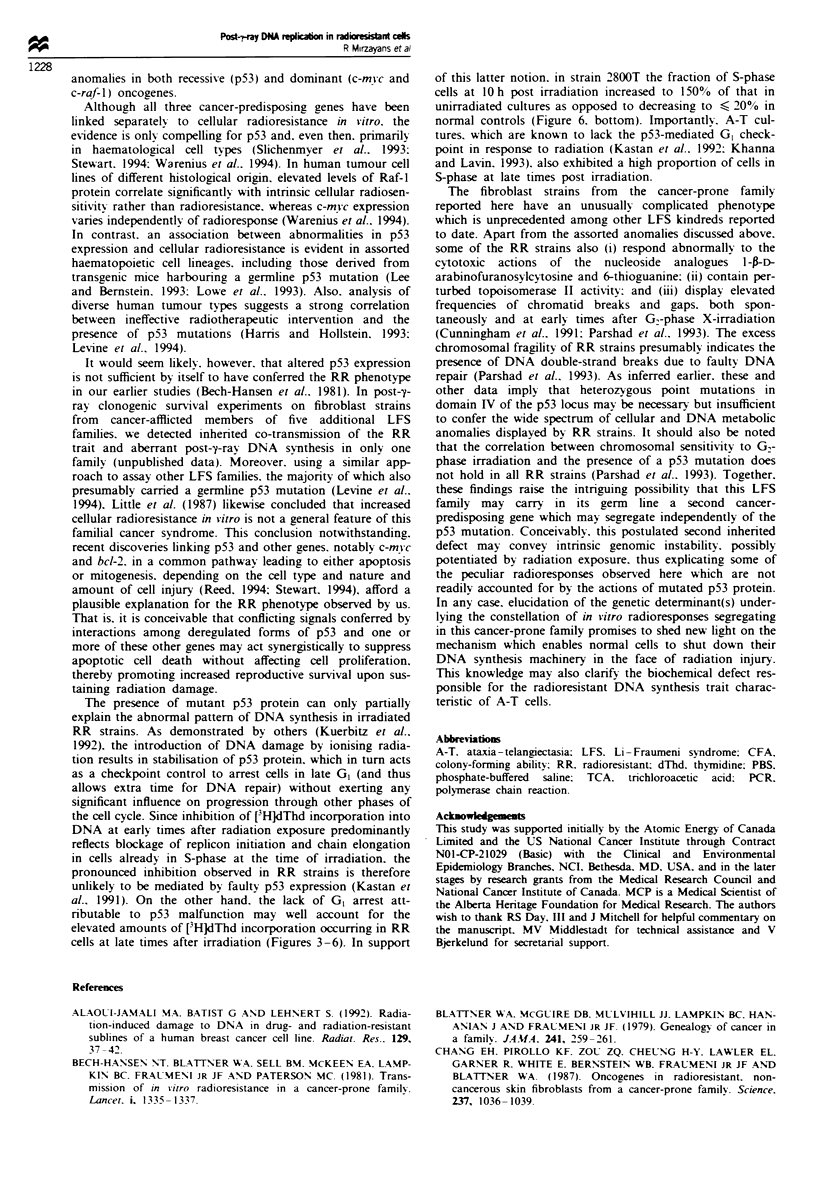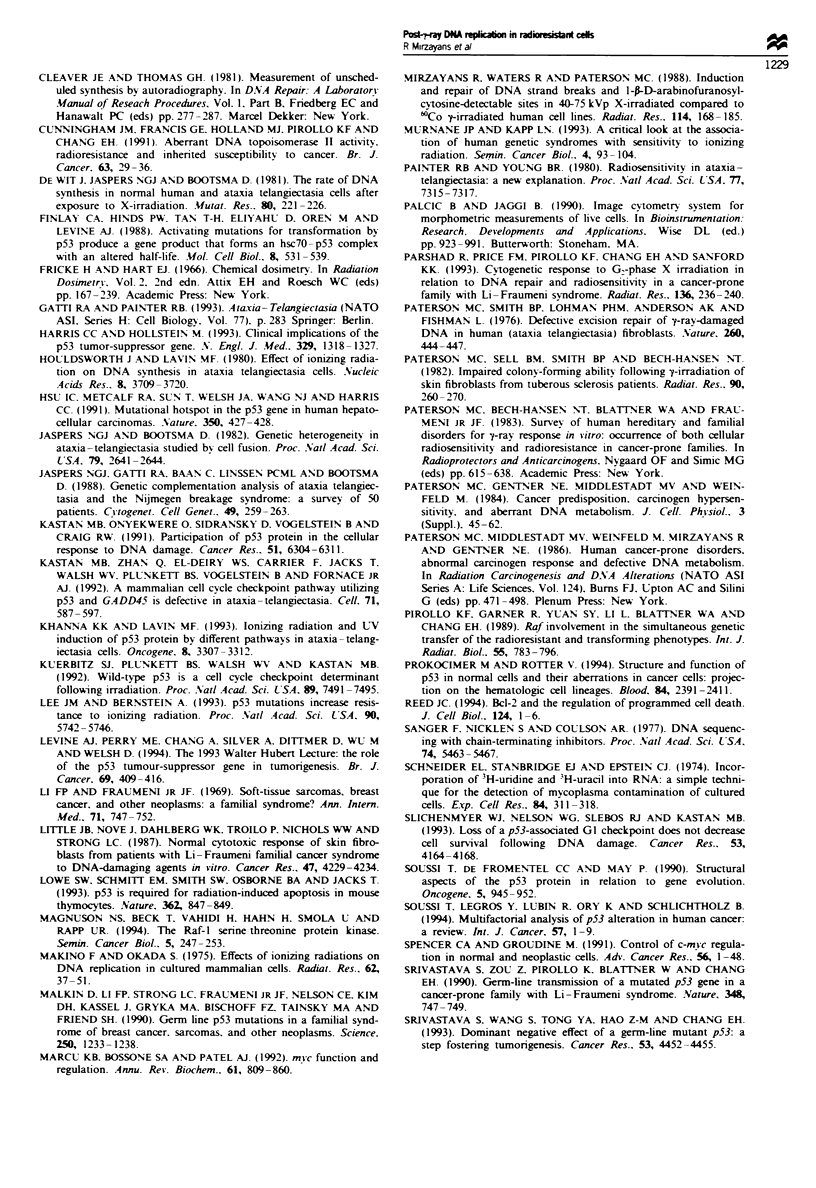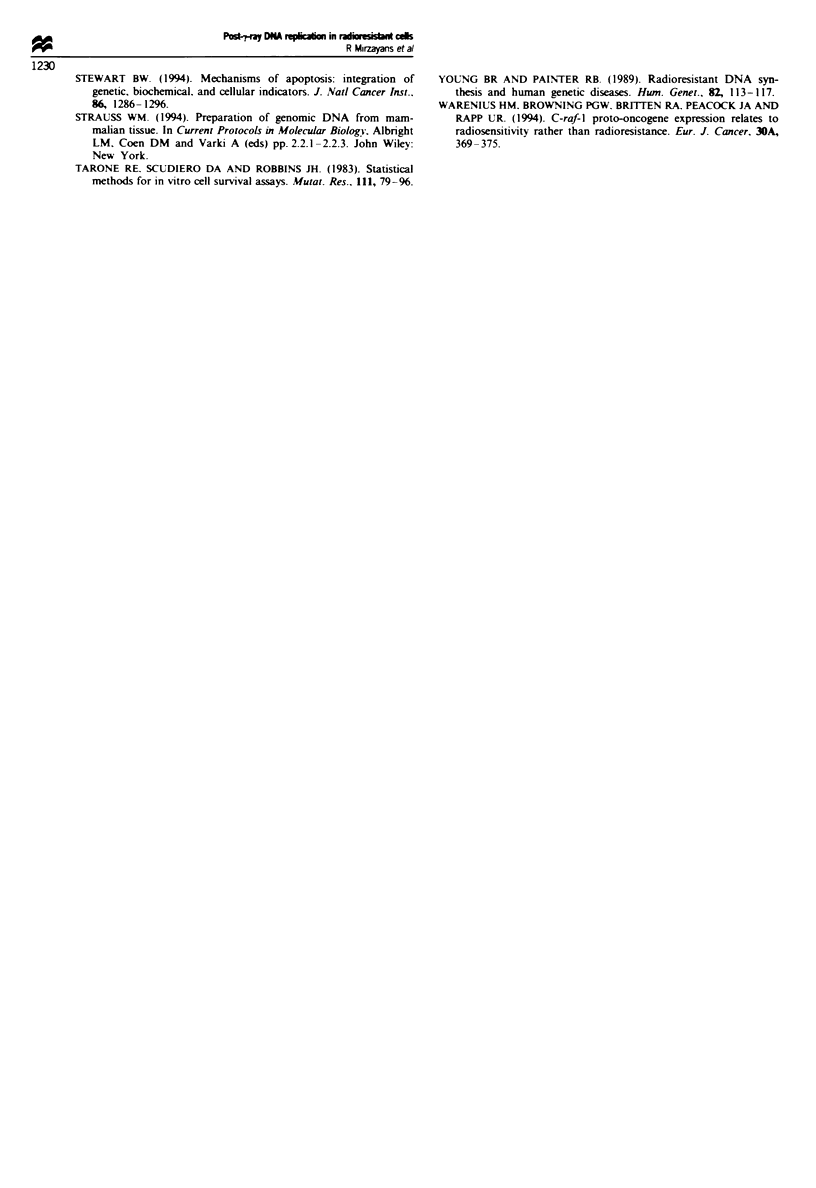# Abnormal pattern of post-gamma-ray DNA replication in radioresistant fibroblast strains from affected members of a cancer-prone family with Li-Fraumeni syndrome.

**DOI:** 10.1038/bjc.1995.237

**Published:** 1995-06

**Authors:** R. Mirzayans, R. A. Aubin, W. Bosnich, W. A. Blattner, M. C. Paterson

**Affiliations:** Molecular Oncology Program, University of Alberta, Edmonton, Canada.

## Abstract

**Images:**


British Journal of Cancer (1995) 71, 1221-1230

© 1995 Stockton Press All rights reserved 0007-0920/95 $12.00

### Abnormal pattern of post-γ-ray DNA replication in radioresistant fibroblast strains from affected members of a cancer-prone family with Li-Fraumeni syndrome

R Mirzayans[1], RA Aubin[2], W Bosnich[1], WA Blattner[3] and MC Paterson[1]

[1]*Molecular Oncology Program, Cross Cancer Institute, and Department of Oncology, University of Alberta, Edmonton, Alberta T6G 1Z2, Canada;* [2]*Health and Welfare Canada, Life Sciences Division - Biotechnology, Sir FG Banting Research Centre, Tunney's Pasture, Ottawa, Ontario K1A 0L2, Canada; and* [3]*Environmental Epidemiology Branch, National Cancer Institute, National Institutes of Health, Bethesda, Maryland 20892, USA.*

**Summary** Non-malignant dermal fibroblast strains, cultured from affected members of a Li-Fraumeni syndrome (LFS) family with diverse neoplasms associated with radiation exposure, display a unique increased resistance to the lethal effects of γ-radiation. In the studies reported here, this radioresistance (RR) trait has been found to correlate strongly with an abnormal pattern of post-γ-ray DNA replicative synthesis, as monitored by radiolabelled thymidine incorporation and S-phase cell autoradiography. In particular, the time interval between the γ-ray-induced shutdown of DNA synthesis and its subsequent recovery was greater in all four RR strains examined and the post-recovery replication rate was much higher and was maintained longer than in normal and spousal controls. Alkaline sucrose sedimentation profiles of pulse-labelled cellular DNA indicated that the unusual pattern of DNA replication in irradiated RR strains may be ascribed to anomalies in both replicon initiation and DNA chain elongation processes. Moreover, the RR strain which had previously displayed the highest post-γ-ray clonogenic survival was found to harbour a somatic (codon 234) mutation (presumably acquired during culture *in vitro*) in the same conserved region of the p53 tumour-suppressor gene as the germline (codon 245) mutation in the remaining three RR strains from other family members, thus coupling the RR phenotype and abnormal post-γ-ray DNA synthesis pattern with faulty p53 expression. Significantly, these two aberrant radioresponse end points, along with documented anomalies in c-*myc* and c-*raf*-1 proto-oncogenes, are unprecedented among other LFS families carrying p53 germline mutations. We thus speculate that this peculiar cancer-prone family may possess in its germ line a second, as yet unidentified, genetic defect in addition to the p53 mutation.

**Keywords:** post-γ-ray DNA replication; radioresistant cells; Li-Fraumeni syndrome; mutated p53 gene; DNA replicons

The recognition of enhanced radiosensitivity as a hallmark of the hereditary neurovascular and cancer-prone disorder ataxia-telangiectasia (A-T) (Gatti and Painter, 1993) has precipitated an intensive search for additional cancer-predisposing conditions associated with radiotoxicity (Murnane and Kapp, 1993). Our ongoing survey of cellular radiation response *in vitro* has focused on clinically affected members of 'cancer families' characterised by a marked excess of histologically proven malignancies, especially those that appear to have arisen on exposure to ionising radiation (Paterson *et al.*, 1983, 1986). In one of the most informative kindreds studied thus far, an aggregation of mesenchymal and epithelial neoplasms representative of those seen in the familial cancer syndrome originally described by Li and Fraumeni (1969) (Li-Fraumeni syndrome, LFS), has appeared over six generations in a pattern compatible with autosomal dominant transmission of an altered, highly penetrant gene (Blattner *et al.*, 1979). Two members of this kindred presented with clinical complications linked to previous radiation exposure: a teenaged boy developed a vertebral osteosarcoma in the field of radiotherapy which had been administered 12 years earlier for a bilateral malignant neurilemmoma; and his paternal great-uncle contracted the preleukaemic condition polycythaemia vera 5 years after occupational exposure to radioactive heavy water (Blattner *et al.*, 1979). Using post-γ-ray colony-forming ability (CFA) as the criterion, a radioresistant (RR) phenotype was observed in cultured non-transformed skin fibroblasts from five (including the aforementioned two) of six family members in the cancer-prone lineage (Bech-Hansen *et al.*, 1981), leading us to hypothesise that such tolerance to the killing effects of radiation may in some way be genetically linked to a propensity to develop a variety of common tumours.

Chang and co-workers reported that RR fibroblast strains from affected members of this LFS kindred exhibit elevated expression of c-*myc* and may, on the basis of indirect evidence, harbour c-*raf*-1 in an activated, tumour-predisposing form (Chang *et al.*, 1987; Pirollo *et al.*, 1989). The products of these two proto-oncogenes are known to participate at different stages in membrane signalling and transduction pathways regulating cell proliferation and apoptosis. c-*raf*-1 is a cytosolic serine threonine protein kinase responsible for transmission of signals initiated at the cell membrane by growth factor receptors and protein kinase C (Magnuson *et al.*, 1994); c-*myc* is a downstream nuclear regulator of gene expression (Spencer and Groudine, 1991; Marcu *et al.*, 1992). In addition, four of the six RR strains, established from different members of this family, carry, in a heterozygous state in the germ line, a point mutation in codon 245 of p53 (Srivastava *et al.*, 1990; Parshad *et al.*, 1993), a tumour-suppressor gene encoding a nuclear phosphoprotein implicated in the control of cell cycle progression via its transcriptional transactivation activity (Levine *et al.*, 1994; Prokocimer and Rotter, 1994).

This study compares the effects of $^{60}$Co γ-rays on the rate of replicative DNA synthesis in several RR and control (normal and A-T) fibroblast strains. We demonstrate that the RR phenotype is accompanied by an abnormal pattern of DNA synthesis following radiation exposure. Evidence is also presented showing that the RR strain, which displays the highest post-γ-ray CFA, harbours a somatic point mutation (presumably acquired during *in vitro* culture) in the same conserved domain of the p53 gene as the germline mutation in other family members, thus coupling these two aberrant radiation response end points with faulty p53 expression.

Correspondence: MC Paterson, Molecular Oncology Program, Department of Experimental Oncology, Cross Cancer Institute, Edmonton, Alberta T6G 1Z2, Canada
Received 20 April 1994; revised 18 January 1995; accepted 26 January 1995

### Materials and methods

### Cell strains and their cultivation

The experiments described below were performed on fibroblast strains established from normal skin biopsy explants of 11 human subjects. Five strains, four from healthy volunteers and one from an A-T patient, served as normal and radiosensitive controls respectively, while the remaining six strains, four from affected members and two from spousal controls in the LFS family, were used as test strains (Table I). Each strain was classified as normal, sensitive or resistant, depending on its CFA status following acute exposure to γ-radiation delivered under oxic conditions. All strains were free of mycoplasma contamination, as judged by assaying exogenous [$^3$H]uridine/[$^3$H]uracil uptake into RNA (Schneider *et al.*, 1974). Cells were cultivated at 37°C in Ham's F12 medium supplemented with 10% (v/v) fetal bovine serum, 1 mM glutamine, 100 units $ml^{-1}$ penicillin G and 100 μg $ml^{-1}$ streptomycin sulphate (henceforth denoted as complete medium) in a humidified atmosphere of 5% carbon dioxide in air. Cell culture supplies were purchased from Gibco (Grand Island, NY, USA).

### Gamma irradiation

Exposure to $^{60}$Co γ-radiation was performed under oxic (air-equilibrated) conditions in a Gammacell 220 Unit (Atomic Energy of Canada Limited, Ottawa, ON, Canada) at a dose rate ranging from 58 to 63 Gy $min^{-1}$, as calibrated by Fricke colorimetry [$G(Fe^{3+}) = 15.6$] (Fricke and Hart, 1966). For low-dose exposure, the dose rate was reduced to 10% or 50% of normal transmission by the use of annular sleeve, cast lead attenuators.

### Measurement of DNA damage and its repair

Cellular DNA was labelled by incubating exponentially growing cultures for 24 h in the presence of either $1.8 \times 10^4$ Bq $ml^{-1}$[methyl-$^3$H]thymidine (dThd) (specific activity, $2.4 \times 10^{11}$ Bq $mmol^{-1}$) or $3.7 \times 10^4$ Bq $ml^{-1}$ [methyl-$^{14}$C]dThd (specific activity, $2 \times 10^9$ Bq $mmol^{-1}$) (New England Nuclear Canada, Lachine, PQ, Canada) in Ham's F12 medium lacking dThd (hereafter referred to as dThd-free medium).

To measure the production and rejoining of γ-ray-induced DNA strand breaks, pairs of RR ([$^3$H]dThd-labelled) and control ([$^{14}$C]dThd-labelled) cultures were trypsinised and co-plated in 60 mm dishes at approximately $10^5$ per dish. Following overnight incubation, the cultures were washed with ice-cold phosphate-buffered saline (PBS) and exposed to a range of γ-ray doses. Immediately thereafter, prewarmed complete medium was added, and the cultures were incubated to allow repair of radiation-induced DNA damage. At suitable times, cell monolayers were scraped into ice-cold PBS (approximately 0.2 ml per dish) and single-cell suspensions prepared. A 50 μl sample of each suspension was then lysed and subjected to alkaline sucrose gradient-velocity sedimentation analysis, as detailed elsewhere (Mirzayans *et al.*, 1988). Analysis of the radioactivity distributions in the gradient profiles yielded the weight-average molecular weight values of the $^3$H- and $^{14}$C-labelled DNAs from which the number of single-strand breaks in genomic DNA was computed.

### Post-irradiation DNA replicative synthesis assays

*Inhibition and recovery measurements* For each strain under study, cultures in late logarithmic growth were seeded at approximately $10^5$ cells per 60 mm dish and incubated overnight in complete medium and for a further 18–20 h in dThd-free medium supplemented with $3.7 \times 10^2$ Bq $ml^{-1}$ [methyl-$^{14}$C]dThd (specific activity, $2 \times 10^9$ Bq $mmol^{-1}$). After removal of the radioactive medium, each culture was incubated in fresh medium for at least 1 h to deplete endogenous DNA precursor pools of residual $^{14}$C-labelled dThd. Cultures were then either exposed to γ-rays or sham irradiated. At specific times during subsequent incubation, the corresponding γ-ray- and sham-treated cell monolayers were pulse labelled for 15 min (unless stated otherwise) in dThd-free medium containing $5.5 \times 10^5$ Bq $ml^{-1}$ [methyl-$^3$H] dThd (specific activity, $3 \times 10^{12}$ Bq $mmol^{-1}$). The paired cultures were immediately lysed, and the lysates were spotted on Whatman No. 17 filters (Fisher Scientific, Toronto, ON, Canada), washed twice in 5% trichloroacetic acid (TCA) and once in 95% ethanol, dried and counted in a liquid scintillation spectrometer (Beckman Instruments, Toronto, ON, Canada). The rate of semiconservative DNA synthesis in

**Table I** Pertinent properties of dermal fibroblast strains and their human (normal, A-T and LFS) donors

| *Strain designation*[a] | *Clinical description at time of biopsy*[b] | *Donor Age*[c] | *Sex* | *Relation* | In vitro *age during study*[d] | *Post-γ-ray CFA phenotype [$D_{10}$ ± s.e. (Gy)]*[e] | *p53 gene status Codon* | *Sequence* |
|---|---|---|---|---|---|---|---|---|
| GM38 | Normal | 9 | Female | | 17–24 | N(4.07 ± 0.15) | 245[f] | GGC |
| GM43 | Normal | 32 | Female | | 19–24 | N(3.82 ± 0.09) | 245[f] | GGC |
| 1387T | Normal | 66 | Male | | 20,23 | N(3.67 ± 0.07) | – | |
| 1461T | Normal | 43 | Male | | 19,21,23 | N(4.17 ± 0.17) | – | |
| AT3BI[g] | A-T | 4 | Male | | 19,23 | S(1.84 ± 0.15) | – | |
| 2675T | Osteosarcoma | 16 | Male | Proband | 19–23 | R(5.02 ± 0.15) | 245[h] | GGC→GAC |
| 1872T | Normal | 32 | Female | Mother/spouse | 22 | N(3.98 ± 0.11) | 245[h] | GGC |
| 1873T | Astrocytoma | 35 | Male | Father | 23 | R(4.80 ± 0.17) | 245[h] | GGC→GAC |
| 2674T | Neurilemmoma, Osteosarcoma | 12 | Male | Brother | 19–23 | R(4.96 ± 0.11) | 245[h] | GGC→GAC |
| 2525T | Normal | 55 | Male | Paternal grandfather spouse | 20,24 | N(4.30 ± 0.09) | 245[h] | GGC |
| 2800T | Polycythaemia vera | 71 | Male | Paternal great uncle | 17–24 | R(5.32 ± 0.17) | 234[f]<br>245[f,i] | TAC→TGC<br>GGC |

[a]The A-T strain was kindly provided by Dr AMR Taylor (University of Birmingham, Birmingham, UK). The GM strains were purchased from the Institute for Medical Research (Camden, NJ, USA). The remainder of the strains were purchased from Meloy Laboratories (Springfield, VA, USA). [b]Since the skin biopsies were taken over a decade ago, new cancers have developed in three of the five LFS family members studied here, namely a primary astrocytoma at age 26 in the proband, a second primary (fatal) brain tumour at age 43 in the proband's father and a fatal colon carcinoma at age 63 in the proband's paternal grandfather, who married into the family and hence did not carry the LFS-predisposing gene (Chang *et al.*, 1987). [c]Age (years) at biopsy. [d]Expressed as cumulative cell population doublings since establishment of the primary fibroblast culture, although stock cultures were usually passaged every 3–6 days at a split ratio of 1:3. [e]Response of indicated strain to the killing effects of $^{60}$Co γ-radiation delivered acutely under oxic (air-equilibrated) conditions. Assignment of each strain to a given class (N, normal; S, sensitive; R, resistant) was determined by using the standard error of the difference between $D_{10}$ values [two-tailed *t*-test of Tarone *et al.* (1983)] as the statistical test and $P < 0.05$ as the criterion of significant difference (for details, see Paterson *et al.*, 1986). Clonogenic survival data were taken from Bech-Hansen *et al.* (1981), Paterson *et al.* (1982, 1983) and our unpublished results. [f]From this paper. [g]Complementation group A (Jaspers *et al.*, 1988). [h]From Srivastava *et al.* (1990). [i]From Parshad *et al.* (1993).

irradiated cultures (expressed as a percentage of that arising in the sham-treated controls) was calculated as follows:

$$\frac{(\text{c.p.m. } ^3\text{H c.p.m. } ^{14}\text{C})_{\text{irradiated}}}{(\text{c.p.m. } ^3\text{H c.p.m. } ^{14}\text{C})_{\text{sham-irradiated}}} \times 100$$

The assay outlined above was performed in two ways. First, a range of doses (≤20 Gy) was delivered and replicative synthesis was determined at a given time (e.g. 30–40 min) during subsequent incubation, thus measuring the degree of synthesis inhibition as a function of radiation dose. Second, a single γ-ray dose (e.g. 10 Gy) was administered, and cultures were pulse labelled after various periods of post-irradiation incubation (≤16 h), hence monitoring both (i) the magnitude of and (ii) the extent and duration of recovery from the transitory depression of DNA synthesis resulting from the radiation treatment.

*Alkaline sucrose gradient analysis* Unlabelled logarithmic cultures were exposed to 10 Gy of γ-radiation (or sham irradiated) and pulse labelled ([methyl-$^3$H]dThd; 15 min) at selected incubation times, as described above. After rinsing with ice-chilled PBS, each culture was mechanically detached, and a 200 μl sample of a single-cell suspension ($10^6$ cells ml$^{-1}$ in PBS) was gently pipetted onto 0.8 ml of lysis solution (1 M sodium hydroxide–0.1 M disodium EDTA) on top of an 11 ml linear gradient of 5–20% (w v) sucrose in 2 M sodium chloride–0.3 M sodium hydroxide–10 mM disodium EDTA (pH 12.5). After holding in the dark at room temperature for 1 h, the gradients were centrifuged (30 000 r.p.m., 3 h, 20°C) in a Sorvall TH-641 rotor driven by a Sorvall RC70 ultracentrifuge (DuPont Canada, Markham, ON, Canada). Finally, each gradient was fractionated (14 drops per fraction), and the TCA-insoluble radioactivity in each fraction was measured. Changes in the shape of the resulting profiles for a particular strain under different treatment conditions and at different times of pulse labelling provided insight into the relative size of nascent DNA strands in S-phase cells as a function of post-irradiation incubation. We were thus able to deduce the inhibitory effect of radiation on (i) initiation of replicating units (replicons) not yet in operation and (ii) chain elongation of replicons already in operation (Painter and Young, 1980).

*Autoradiography* The rate of DNA synthesis per S-phase cell was determined by *in situ* autoradiography. Logarithmic phase cultures were seeded on sterile glass cover slips (placed in 35 mm dishes) at approximately $5 \times 10^4$ cells in a final volume of 2 ml of dThd-free medium. After incubation for 2 days, cultures were exposed to 10 Gy of γ-rays (or sham irradiated), incubated for 1 or 2 h in dThd-free medium, and then pulse labelled for 30 min in medium containing $3.7 \times 10^4$ Bq [methyl-$^3$H]dThd (specific activity, $2.4 \times 10^{11}$ Bq mmol$^{-1}$) per ml (Jaspers and Bootsma, 1982). Cells were rinsed with PBS and incubated for 10 min with diluted fixing solution [methanol acetic acid (3:1), mixed 1:1 in PBS], followed by incubation with undiluted fixing solution for a further 10 min, before drying in air. Cover slips were mounted on glass microscope slides, which were then dipped in Kodak NTB-2 nuclear track emulsion, dried, exposed at 4°C for 7 days, and finally processed in a Kodak D19 developer (Cleaver and Thomas, 1981). The number of silver grains above the nucleus of S-phase cells was determined automatically by digitised image microscopy (Palcic and Jaggi, 1990).

The protocol for measuring the fraction of cells in S-phase following irradiation was identical to that described above, except that cultures were pulse labelled with high specific activity ($3 \times 10^{12}$ Bq mmol$^{-1}$) [$^3$H]dThd at a concentration of $3.7 \times 10^5$ Bq ml$^{-1}$.

*p53 gene sequencing*

Genomic DNA of strains GM38, 2674T and 2800T (approximately $10^6$ cells per strain) was isolated by proteinase K–sodium dodecyl sulphate digestion, deproteinisation with successive phenol–chloroform–isoamyl alcohol extractions and ethanol precipitation (Strauss, 1994). Using the combination of external [i.e. polymerase chain reaction (pcr)] and internal [i.e. sequencing (seq)] primer sets described by Hsu *et al.* (1991), exons 5–8 of the p53 gene were sequenced after amplification by polymerase chain reaction (PCR). For each PCR amplification, four reactions were required to obtain sufficient product. In each reaction 0.25 μg of cellular DNA was incubated in 100 μl of solution containing 10 mM potassium chloride, 10 mM ammonium sulphate, 20 mM Tris–HCl (pH 8.8 at 25°C), 2 mM magnesium sulphate, 0.1% Triton X-100, 100 pmol of oligonucleotide primers, 1.5 mM dNTPs (Pharmacia Biotech, Piscataway, NJ, USA) and 2 units of Vent DNA polymerase (New England Biolabs, Beverly, MA, USA). Each template was denatured for 5 min at 96°C, followed by 37 cycles of PCR in a Thermal Reactor II unit (Tyler Research Instruments, Edmonton, AB, Canada) with incubations for 20 s at 96°C (denaturation), 35 s at 55°C (annealing) and 40 s at 73°C (extension), and a final extension step for 5 min at 73°C. The four PCR reaction products were combined and purified by acrylamide (12%) gel electrophoresis, electroelution, phenol– chloroform extraction and ethanol precipitation. The final purified products were sequenced by a modification of the dideoxy chain-termination method of Sanger *et al.* (1977) according to the T7 Sequencing Kit (Pharmacia). Each reaction contained 1 pmol of sequencing primers, 5 units of T7 DNA polymerase and $3.7 \times 10^5$ Bq of [α-$^{35}$S]dATPαS (specific activity, $42 \times 10^{12}$ Bq mmol$^{-1}$). Finally, the sequencing reaction products were subjected to electrophoresis in denaturing polyacrylamide (8%) gels followed by autoradiography.

### Results

*Induction and repair of DNA radioproducts*

RR and normal strains were compared with respect to the initial yield and subsequent restitution of strand breaks. Strain 2800T, derived from the proband's paternal great-uncle with suspected radiation-induced disease, displayed the highest post-γ-ray CFA in our original report (Bech-Hansen *et al.*, 1981; see Table I) and was therefore chosen as the representative RR strain in the first set of experiments. As depicted in Figure 1, strand breaks were produced in γ-irradiated normal (1387T) and RR (2800T) strains as a linear function of the dose (10–200 Gy) administered. The strand

**Figure 1** Induction and rejoining of DNA single-strand breaks in normal control (1387T; open symbols) and RR (2800T; closed symbols) fibroblast strains after exposure to $^{60}$Co γ-radiation. Prelabelled cultures were treated with various doses of γ-rays, and their DNA was sedimented in alkaline sucrose gradients either immediately (diamonds) or following cell incubation in complete medium for 1.5 min (circles) or 6 min (triangles) (left) or for 15 min (circles), 30 min (triangles) or 45 min (squares) (right).

breakage incidence was the same for both strains, i.e. ~3.6 breaks $10^{-10}$ daltons $Gy^{-1}$. Likewise, upon incubation of the cell cultures following irradiation, strand breaks were found to disappear in the two strains with very similar kinetics (Figure 1). In both strains, approximately 85% of the breaks introduced by 10 Gy and 100 Gy were rejoined within approximately 6 min and approximately 30 min respectively. The results obtained with additional test (RR and spousal control) and normal control strains following exposure to a single dose (100 Gy) of γ-rays are shown in Figure 2. As was seen in 2800T cells, strand breaks were introduced and removed at normal rates in the RR strains 1873T, 2674T and 2675T.

### *Inhibition of DNA synthesis by γ-rays*

The effects of different γ-ray doses on DNA replicative synthesis in strain 2800T and in normal (GM38 and 1387T) and radiosensitive (AT3BI) controls are illustrated in Figure 3. In the two normal strains, the rate of DNA synthesis at early times after irradiation (top) decreased in a biphasic manner over the dose range (0–20 Gy) examined, declining sharply at lower doses (e.g. approximately 75% of the control rate at 5 Gy) and then more gradually at higher doses (e.g. approximately 60% of the control level at 20 Gy). Such biphasic dose–response curves are characteristic of mammalian cells in general and human cells in particular; hence our results conform with earlier ones (see, for example, Houldsworth and Lavin, 1980; Young and Painter, 1989). As expected (Young and Painter, 1989), AT3BI cells exhibited a striking attenuation in the suppression of DNA synthesis by γ-rays, carrying out dThd incorporation at approximately 80% of the untreated value even after receiving a supralethal dose of 20 Gy (Figure 3). In keeping with the findings of others (Houldsworth and Lavin, 1980; de Wit *et al.*, 1981), the dose–response curve for the A-T strain was monophasic with a slope roughly parallel to that of the shallow, high-dose component of the curves for the two normal strains. As shown in the lower panel of Figure 3, the abnormally high rate of DNA synthesis in A-T cells was maintained at 10 h post irradiation.

It is apparent in Figure 3 that exposure of 2800T cells to γ-radiation caused an early inhibition of DNA synthesis which was more depressed than that observed in irradiated normal cells. Interestingly, the dose–response pattern exhibited by these radioresistant cells was in the opposite direction from normal with respect to that displayed by the radiosensitive A-T cells during the 15 to 75 min post-γ-ray labelling period, that is DNA synthesis was suppressed to a greater extent in RR cells than in normal controls at each dose administered [e.g. 50% *vs* approximately 65% of the control DNA synthesis level, respectively, after 10 Gy (Figure 3, top)]. Furthermore, the data in the lower panel of Figure 3 suggest that 2800T cells possess a markedly greater potential than do normal cells of recovering from the inhibitory effects of γ-rays on DNA synthesis, as the rate of [$^3$H]dThd incorporation in 2800T cells rose through the normal range to the level occurring in A-T cells by 10 h following irradiation.

### *Kinetics of DNA synthesis during post-irradiation cell incubation*

To follow the time course of change in post-γ-ray DNA synthesis, cultures were exposed to a dose of 10 Gy and pulse labelled with [$^3$H]dThd at various times during subsequent incubation. The outcome of a typical experiment involving 2800T and two normal control strains is depicted in Figure 4, and results from multiple experiments on several test (RR and spousal control) and control (normal and A-T) strains are summarised in Figure 5; 2800T and GM38 were included in all experiments as reference strains. In all four normal controls, the replication rate decreased sharply, reaching a minimal level, namely approximately 55% of that of the sham-irradiated samples, at 1 h post treatment (Figure 5, top left); this was followed by a 3 h recovery phase whereupon synthesis again declined rapidly such that by 8 h the level was

**Figure 2** Induction and rejoining of DNA single-strand breaks in the indicated [$^{14}$C]dThd-labelled control (spousal and normal) (○) and [$^3$H]dThd-labelled RR (●) strains. Each pair of control and RR cultures was γ-irradiated (100 Gy) and, after co-incubation for various times, their DNA was co-sedimented in alkaline sucrose gradients. Data represent the mean (± range) of duplicate independent determinations.

**Figure 3** Rate of DNA synthesis (measured by [$^3$H]dThd incorporation) in normal control (GM38 and 1387T), RR (2800T) and radiosensitive control (AT3BI) strains after receiving 2.5–20 Gy of γ-radiation. Cultures were pulse labelled with [$^3$H]dThd for 60 min beginning at 15 min (top) or 9.5 h (bottom) after irradiation. Each datum point represents the mean (± s.e.) of triplicate independent determinations.

only 40% of that occurring in undamaged cultures. As is evident in Figure 4 and the lower left panel of Figure 5, the kinetics of post-irradiation DNA synthesis in 2800T cells differed from that arising in normal controls in the following ways: (i) the initial depression was both more abrupt and more extensive, falling to approximately 45%, compared with approximately 55%, of the unirradiated value; (ii) the time at which recovery began was delayed by about 1 h, commencing at approximately 2 h after irradiation; (iii) the extent of increase during the recovery phase (i.e. the difference between minimal and maximal levels) was more than 2-fold greater

**Figure 4** Kinetics of [$^3$H]dThd incorporation into DNA of normal control (GM38, GM43) and RR (2800T) fibroblast strains after exposure to 10 Gy of γ-rays. Cells were pulse labelled with [$^3$H]dThd for 15 min beginning at indicated times after irradiation. Each datum point represents the mean (± s.e.) of triplicate samples.

(approximately 50% *vs* approximately 25%); and (iv) the high level of post-recovery replication was sustained much longer.

The responses of the RR strains 1873T, 2674T and 2675T proved to be very similar to that described above for strain 2800T, except that 1873T cells consistently exhibited normal levels of DNA synthesis inhibition at early times ($\leqslant 1$ h) after irradiation (Figure 5, upper right). The differences between the values obtained for normal strains and each of the four RR strains at 1.5, 2 and 8 h post irradiation were highly significant (*P*-values $\leqslant 0.005$), as determined by conventional Student's *t*-test analysis. As expected, the spousal control strains 1872T and 2525T responded normally to the disruptive effect of γ-rays on DNA synthesis (Figure 5, top middle). Quantitatively similar results were obtained when the kinetics of DNA synthesis was compared in representative RR (e.g. 2800T, 1873T) and normal and spousal control (e.g. GM38, 2525T) strains following exposure to lower doses (2.5 and 5 Gy) of γ-radiation (data not shown).

### *Effects of γ-rays on rate of DNA synthesis per S-phase cell and on fraction of cells in S-phase*

Using *in situ* autoradiography, we compared the rate of DNA synthesis in S-phase cells and the fraction of S-phase cells in RR (2800T), normal control (GM43), spousal control (2525T) and radiosensitive (AT3BI) cultures. In each strain, the kinetics of DNA synthesis per S-phase cell measured at early times after γ-irradiation (Figure 6, top) was similar to that seen in an asynchronously dividing culture by scintillation counting (Figures 4 and 5). Thus, as expected (Khanna and Lavin, 1993), the rate of [$^3$H]dThd incorporation into genomic DNA at short intervals post irradiation primarily reflected DNA synthesis in cells already in S-phase at the time of radiation exposure. The fraction of cells in S-phase at $\leqslant 2$ h following irradiation was similar to that in sham-treated cultures (Figure 6, bottom). At later times, however, the S-phase fraction decreased in a time-dependent manner in

**Figure 5** Kinetics of [$^3$H]dThd incorporation into DNA of fibroblast strains from four clinically normal volunteers, six LFS family members (four RR and two spousal controls) and one A-T patient (AT3BI). Data represent the mean (± s.e.) of 3–7 independent experiments. The broken line denotes the mean (± s.e.) of the values obtained with the four normal control strains in the upper left panel.

**Figure 6** Rate of DNA synthesis per S-phase cell (top) and fraction of cells in S-phase (bottom) in the indicated fibroblast strains at various times after exposure to γ-rays (10 Gy). Top: Following irradiation, cultures were incubated in growth medium for indicated times and then pulse labelled for 30 min with low specific activity [$^3$H]dThd ($2.4 \times 10^{11}$ Bq mmol$^{-1}$; $3.7 \times 10^4$ Bq ml$^{-1}$). The rate of DNA synthesis per S-phase cell was determined by *in situ* autoradiography as described in Materials and methods. Each datum point represents the mean (± s.e.) of number of grains above ≥ 200 S-phase cells in duplicate samples. Bottom: Cultures were γ-irradiated, incubated in growth medium and then pulse labelled for 30 min with high specific activity [$^3$H]dThd ($3 \times 10^{12}$ Bq mmol$^{-1}$; $3.7 \times 10^5$ Bq ml$^{-1}$). Cells were processed for autoradiography and the fraction of cells in S-phase (i.e. those with a heavily labelled nucleus) was determined. The data were normalised such that 100% corresponds to the fraction of S-phase cells in control (sham-irradiated) cultures. Each datum point represents the mean (± range) of the values obtained in duplicate samples.

the two normal strains but increased in 2800T and AT3BI cells. As will be elaborated on in the Discussion, these results indicate the absence of a normal cell cycle ($G_1$ phase) arrest in response to γ-rays in both RR and A-T cells.

### *Effects of γ-rays on DNA replicon initiation and chain elongation*

To investigate whether the altered kinetics of DNA synthesis in RR cells may be due to an anomaly in radiation-induced arrest of replicon initiation and/or chain elongation, we carried out an alkaline sucrose gradient-velocity sedimentation analysis of nascent DNA strands formed in cells at various times after exposure to 10 Gy of γ-rays. The resulting radioactivity profiles of DNA from control (i.e. sham-irradiated) cultures of both normal (GM38) and RR (2800T) cells contained two distinct regions (Figure 7, left): a low molecular weight component (fractions 12–26) reflecting DNA molecules that were initiating replication during the pulse-labelling period and a high molecular weight region (fractions 4–11) depicting preinitiated molecules that were undergoing chain elongation and joining with their neighbours during the same pulse-labelling period (Makino and Okada, 1975; Painter and Young, 1980). When the normal and RR fibroblasts were irradiated and then incubated for 2 h before pulse labelling, their sedimentation profiles contained decreased amounts of radioactivity compared with that found in the profiles of the corresponding control cultures, and this reduced uptake was found across the entire gradient. In GM38 cells the radiation treatment produced a preferential suppression of precursor incorporation into smaller sized DNA species. This is reminiscent of results reported by others for various mammalian cell lines (Makino and Okada, 1975; Painter and Young, 1980), and is interpreted to indicate that the inhibition of DNA synthesis by moderate doses of ionising radiation stems predominantly from blockage of replicon initiation (Painter and Young, 1980). The reduction of radioactivity in both the low and high molecular weight regions was significantly greater in irradiated 2800T (approximately 35 and 50% of unirradiated respectively) than in irradiated GM38 cells (approximately 53 and 68% of unirradiated respectively). However, at 10 h post irradiation the situation was reversed. In the normal cells the amount of radioactivity in the two regions was depressed much farther than seen at the earlier time, whereas in the RR cells the radioactivity levels in both regions had recovered to that arising in sham-irradiated cultures. Qualitatively similar results were obtained when the responses of RR strains 2674T and 1873T were compared with those of normal (GM43) and spousal (1872T) controls respectively (Figure 7).

Subsequently, we generated alkaline sucrose sedimentation profiles of DNA from GM43, 1872T and 2800T that had been pulse labelled at different intervals (0.5, 1, 2, 3 and 4 h) after exposure to 10 Gy (data not shown). In all instances, the differences in the extent of DNA synthesis inhibition between 2800T and the two normal strains were found to be similar for high *vs* low molecular weight DNA fragments. The data therefore imply that the abnormal kinetics of post-irradiation DNA synthesis displayed by RR cells (Figures 3–6) is a direct manifestation of alterations in both replicon initiation and chain elongation.

### *Status of p53 gene in 2800T cells*

As indicated earlier, 2800T is the most radioresistant strain in the LFS family and has been the subject of several reports associating increased radiation tolerance with germline transmission of a point mutation in codon 245 (G<u>G</u>C→G<u>A</u>C; glycine→aspartic acid) of the p53 gene in this kindred (Chang *et al.*, 1987; Pirollo *et al.*, 1989; Cunningham *et al.*, 1991). Nevertheless, with the exception of a reported normal codon 245 (Parshad *et al.*, 1993), strain 2800T has apparently not been analysed for mutations in the p53 locus. We thus used PCR to amplify and sequence exons 5, 6, 7 and 8 encompassing highly conserved domains III, IV and V (Soussi *et al.*, 1990), as these regions are well-known mutational hotspots in the p53 gene (Harris and Hollstein, 1993; Levine *et al.*, 1994; Soussi *et al.*, 1994). 2800T cells do indeed harbour the wild-type codon 245 of p53 (Figure 8). However, an A→G transition mutation was detected in these same cells at the second nucleotide of codon 234 in the non-coding strand of p53. This point mutation, which results in cysteine replacing tyrosine at this position, was verified by sequencing the coding strand of the product as well as both the coding and non-coding strands of two other PCR samples. No additional mutations were observed in exons 5, 6 or 8 or elsewhere in codon 7 (unpublished data). As illustrated in Figure 8, 2800T fibroblasts also contain the wild-type sequence, TAC, for codon 234, signifying the presence of both wild-type and mutated p53 alleles in these RR cells. Since the nucleotide substitution in codon 234 was not present in the DNA of cultured cells from other family members (data not shown), this point mutation was presumably acquired by 2800T fibroblasts during cultivation *in vitro*. Similar p53 gene mutations have been reported to arise in other cell types during long-term culture (unpublished findings cited in Malkin *et al.*, 1990).

**Figure 7** Alkaline sucrose sedimentation profiles of DNA from normal and spousal control (top) and RR (bottom) fibroblast cultures that were pulse labelled with [$^3$H]dThd for 15 min beginning at 2 h (○) or 10 h (△) after exposure to 10 Gy of γ-radiation. Sedimentation profiles of DNA from sham-treated, pulse-labelled cultures are denoted by closed circles. Each profile represents the mean of three gradients. Sedimentation was from right to left.

**Figure 8** Sequencing autoradiogram of the p53 gene for the non-coding strand in the region of domain IV for PCR-amplified DNA samples from GM38 (normal control) and 2800T (RR) cells. Both mutated cytosine and normal thymine bands at position 2 of codon 234 can be seen. Consult Materials and methods for experimental details. The pcr and seq primers used were those for exon 7 as given in Hsu *et al.* (1991).

### Discussion

We reported previously that non-transformed fibroblast strains from afflicted members of an LFS kindred prone to radiation-induced malignancies exhibit increased resistance to reproductive inactivation by ionising radiation (Bech-Hansen *et al.*, 1981). Follow-up studies, including those presented here, have provided a general framework for delineating the DNA metabolic anomalies co-segregating with the *in vitro* RR phenotype in this family. RR strains excise base and sugar radioproducts and perform γ-ray-induced DNA repair replication normally (Paterson *et al.*, 1983). Likewise, radiation-induced DNA strand breaks are both formed and rejoined at normal rates in these same strains (Figures 1 and 2). Thus, the RR trait is unlikely to be a consequence of either a dose attenuation factor (e.g. increased levels of glutathione or another free radical scavenger; Alaoui-Jamali *et al.*, 1992) or a hyperactive DNA repair system (Paterson *et al.*, 1976, 1984). However, as shown in Figures 3–5, all four RR strains examined exhibit an abnormal pattern of DNA synthesis upon receiving moderate dose radiation. In particular, the interval before recovery from γ-ray-induced inhibition of DNA synthesis is greater in RR than in normal fibroblasts. Also, the level of the post-recovery [$^3$H]dThd incorporation in RR strains is more extensive and the recovery is maintained much longer than that in normal controls. This marked reduction in DNA precursor uptake at earlier times after irradiation, which is also seen as reduced silver grain counts on autoradiograms of S-phase RR cells (Figure 6), can be ascribed to an excessive and sustained shutdown of the DNA synthesis apparatus, with respect to both initiating new replicons and extending those already in operation (Figure 7).

In view of the co-occurrence of aberrant DNA synthesis and enhanced clonogenic survival in RR strains post irradiation, it is tempting to speculate that both irregularities have a common genetic basis which also predisposes to cancer in this LFS kindred. In this regard, the reported presence of c-*myc* and possibly c-*raf*-1 in deregulated states in two RR strains (2800T and 2675T) (Chang *et al.*, 1987; Pirollo *et al.*, 1989) is noteworthy. So too is our demonstration that 2800T cells contain a missense mutation within the same conserved region (domain IV spanning codons 234–258; Soussi *et al.*, 1990) of the p53 gene (Figure 8) as the germline mutation in the remaining three RR strains studied here (Srivastava *et al.*, 1990; see Table I). In the last three RR strains, the mutant and wild-type p53 gene products, although present in equal amounts, combine to form a severely impaired tetrameric protein complex (Srivastava *et al.*, 1993). The somatic mutation in 2800T cells might be expected to convey a similar dominant-negative effect on p53 function (Finlay *et al.*, 1988). In short, at least two of the RR strains may harbour anomalies in both recessive (p53) and dominant (c-*myc* and c-*raf*-1) oncogenes.

Although all three cancer-predisposing genes have been linked separately to cellular radioresistance *in vitro*, the evidence is only compelling for p53 and, even then, primarily in haematological cell types (Slichenmyer *et al*., 1993; Stewart, 1994; Warenius *et al*., 1994). In human tumour cell lines of different histological origin, elevated levels of Raf-1 protein correlate significantly with intrinsic cellular radiosensitivity rather than radioresistance, whereas c-*myc* expression varies independently of radioresponse (Warenius *et al*., 1994). In contrast, an association between abnormalities in p53 expression and cellular radioresistance is evident in assorted haematopoietic cell lineages, including those derived from transgenic mice harbouring a germline p53 mutation (Lee and Bernstein, 1993; Lowe *et al*., 1993). Also, analysis of diverse human tumour types suggests a strong correlation between ineffective radiotherapeutic intervention and the presence of p53 mutations (Harris and Hollstein, 1993; Levine *et al*., 1994).

It would seem likely, however, that altered p53 expression is not sufficient by itself to have conferred the RR phenotype in our earlier studies (Bech-Hansen *et al*., 1981). In post-γ-ray clonogenic survival experiments on fibroblast strains from cancer-afflicted members of five additional LFS families, we detected inherited co-transmission of the RR trait and aberrant post-γ-ray DNA synthesis in only one family (unpublished data). Moreover, using a similar approach to assay other LFS families, the majority of which also presumably carried a germline p53 mutation (Levine *et al*., 1994), Little *et al*. (1987) likewise concluded that increased cellular radioresistance *in vitro* is not a general feature of this familial cancer syndrome. This conclusion notwithstanding, recent discoveries linking p53 and other genes, notably c-*myc* and *bcl*-2, in a common pathway leading to either apoptosis or mitogenesis, depending on the cell type and nature and amount of cell injury (Reed, 1994; Stewart, 1994), afford a plausible explanation for the RR phenotype observed by us. That is, it is conceivable that conflicting signals conferred by interactions among deregulated forms of p53 and one or more of these other genes may act synergistically to suppress apoptotic cell death without affecting cell proliferation, thereby promoting increased reproductive survival upon sustaining radiation damage.

The presence of mutant p53 protein can only partially explain the abnormal pattern of DNA synthesis in irradiated RR strains. As demonstrated by others (Kuerbitz *et al*., 1992), the introduction of DNA damage by ionising radiation results in stabilisation of p53 protein, which in turn acts as a checkpoint control to arrest cells in late $G_1$ (and thus allows extra time for DNA repair) without exerting any significant influence on progression through other phases of the cell cycle. Since inhibition of [$^3$H]dThd incorporation into DNA at early times after radiation exposure predominantly reflects blockage of replicon initiation and chain elongation in cells already in S-phase at the time of irradiation, the pronounced inhibition observed in RR strains is therefore unlikely to be mediated by faulty p53 expression (Kastan *et al*., 1991). On the other hand, the lack of $G_1$ arrest attributable to p53 malfunction may well account for the elevated amounts of [$^3$H]dThd incorporation occurring in RR cells at late times after irradiation (Figures 3–6). In support of this latter notion, in strain 2800T the fraction of S-phase cells at 10 h post irradiation increased to 150% of that in unirradiated cultures as opposed to decreasing to ⩽ 20% in normal controls (Figure 6, bottom). Importantly, A-T cultures, which are known to lack the p53-mediated $G_1$ checkpoint in response to radiation (Kastan *et al*., 1992; Khanna and Lavin, 1993), also exhibited a high proportion of cells in S-phase at late times post irradiation.

The fibroblast strains from the cancer-prone family reported here have an unusually complicated phenotype which is unprecedented among other LFS kindreds reported to date. Apart from the assorted anomalies discussed above, some of the RR strains also (i) respond abnormally to the cytotoxic actions of the nucleoside analogues 1-β-D-arabinofuranosylcytosine and 6-thioguanine; (ii) contain perturbed topoisomerase II activity; and (iii) display elevated frequencies of chromatid breaks and gaps, both spontaneously and at early times after $G_2$-phase X-irradiation (Cunningham *et al*., 1991; Parshad *et al*., 1993). The excess chromosomal fragility of RR strains presumably indicates the presence of DNA double-strand breaks due to faulty DNA repair (Parshad *et al*., 1993). As inferred earlier, these and other data imply that heterozygous point mutations in domain IV of the p53 locus may be necessary but insufficient to confer the wide spectrum of cellular and DNA metabolic anomalies displayed by RR strains. It should also be noted that the correlation between chromosomal sensitivity to $G_2$-phase irradiation and the presence of a p53 mutation does not hold in all RR strains (Parshad *et al*., 1993). Together, these findings raise the intriguing possibility that this LFS family may carry in its germ line a second cancer-predisposing gene which may segregate independently of the p53 mutation. Conceivably, this postulated second inherited defect may convey intrinsic genomic instability, possibly potentiated by radiation exposure, thus explicating some of the peculiar radioresponses observed here which are not readily accounted for by the actions of mutated p53 protein. In any case, elucidation of the genetic determinant(s) underlying the constellation of *in vitro* radioresponses segregating in this cancer-prone family promises to shed new light on the mechanism which enables normal cells to shut down their DNA synthesis machinery in the face of radiation injury. This knowledge may also clarify the biochemical defect responsible for the radioresistant DNA synthesis trait characteristic of A-T cells.

### Abbreviations

A-T, ataxia-telangiectasia; LFS, Li–Fraumeni syndrome; CFA, colony-forming ability; RR, radioresistant; dThd, thymidine; PBS, phosphate-buffered saline; TCA, trichloroacetic acid; PCR, polymerase chain reaction.

### Acknowledgements

This study was supported initially by the Atomic Energy of Canada Limited and the US National Cancer Institute through Contract N01-CP-21029 (Basic) with the Clinical and Environmental Epidemiology Branches, NCI, Bethesda, MD, USA, and in the later stages by research grants from the Medical Research Council and National Cancer Institute of Canada. MCP is a Medical Scientist of the Alberta Heritage Foundation for Medical Research. The authors wish to thank RS Day, III and J Mitchell for helpful commentary on the manuscript, MV Middlestadt for technical assistance and V Bjerkelund for secretarial support.

### References

ALAOUI-JAMALI MA, BATIST G AND LEHNERT S. (1992). Radiation-induced damage to DNA in drug- and radiation-resistant sublines of a human breast cancer cell line. *Radiat. Res.*, **129**, 37–42.

BECH-HANSEN NT, BLATTNER WA, SELL BM, McKEEN EA, LAMPKIN BC, FRAUMENI JR JF AND PATERSON MC. (1981). Transmission of *in vitro* radioresistance in a cancer-prone family. *Lancet*, **i**, 1335–1337.

BLATTNER WA, McGUIRE DB, MULVIHILL JJ, LAMPKIN BC, HANANIAN J AND FRAUMENI JR JF. (1979). Genealogy of cancer in a family. *JAMA*, **241**, 259–261.

CHANG EH, PIROLLO KF, ZOU ZQ, CHEUNG H-Y, LAWLER EL, GARNER R, WHITE E, BERNSTEIN WB, FRAUMENI JR JF AND BLATTNER WA. (1987). Oncogenes in radioresistant, noncancerous skin fibroblasts from a cancer-prone family. *Science*, **237**, 1036–1039.

CLEAVER JE AND THOMAS GH. (1981). Measurement of unscheduled synthesis by autoradiography. In *DNA Repair: A Laboratory Manual of Reseach Procedures*, Vol. 1, Part B, Friedberg EC and Hanawalt PC (eds) pp. 277-287. Marcel Dekker: New York.

CUNNINGHAM JM, FRANCIS GE, HOLLAND MJ, PIROLLO KF AND CHANG EH. (1991). Aberrant DNA topoisomerase II activity, radioresistance and inherited susceptibility to cancer. *Br. J. Cancer*, **63**, 29-36.

DE WIT J, JASPERS NGJ AND BOOTSMA D. (1981). The rate of DNA synthesis in normal human and ataxia telangiectasia cells after exposure to X-irradiation. *Mutat. Res.*, **80**, 221-226.

FINLAY CA, HINDS PW, TAN T-H, ELIYAHU D, OREN M AND LEVINE AJ. (1988). Activating mutations for transformation by p53 produce a gene product that forms an hsc70-p53 complex with an altered half-life. *Mol. Cell Biol.*, **8**, 531-539.

FRICKE H AND HART EJ. (1966). Chemical dosimetry. In *Radiation Dosimetry*, Vol. 2, 2nd edn. Attix EH and Roesch WC (eds) pp. 167-239. Academic Press: New York.

GATTI RA AND PAINTER RB. (1993). *Ataxia-Telangiectasia* (NATO ASI, Series H: Cell Biology, Vol. 77), p. 283 Springer: Berlin.

HARRIS CC AND HOLLSTEIN M. (1993). Clinical implications of the p53 tumor-suppressor gene. *N. Engl. J. Med.*, **329**, 1318-1327.

HOULDSWORTH J AND LAVIN MF. (1980). Effect of ionizing radiation on DNA synthesis in ataxia telangiectasia cells. *Nucleic Acids Res.*, **8**, 3709-3720.

HSU IC, METCALF RA, SUN T, WELSH JA, WANG NJ AND HARRIS CC. (1991). Mutational hotspot in the p53 gene in human hepatocellular carcinomas. *Nature*, **350**, 427-428.

JASPERS NGJ AND BOOTSMA D. (1982). Genetic heterogeneity in ataxia-telangiectasia studied by cell fusion. *Proc. Natl Acad. Sci. USA*, **79**, 2641-2644.

JASPERS NGJ, GATTI RA, BAAN C, LINSSEN PCML AND BOOTSMA D. (1988). Genetic complementation analysis of ataxia telangiectasia and the Nijmegen breakage syndrome: a survey of 50 patients. *Cytogenet. Cell Genet.*, **49**, 259-263.

KASTAN MB, ONYEKWERE O, SIDRANSKY D, VOGELSTEIN B AND CRAIG RW. (1991). Participation of p53 protein in the cellular response to DNA damage. *Cancer Res.*, **51**, 6304-6311.

KASTAN MB, ZHAN Q, EL-DEIRY WS, CARRIER F, JACKS T, WALSH WV, PLUNKETT BS, VOGELSTEIN B AND FORNACE JR AJ. (1992). A mammalian cell cycle checkpoint pathway utilizing p53 and *GADD45* is defective in ataxia-telangiectasia. *Cell*, **71**, 587-597.

KHANNA KK AND LAVIN MF. (1993). Ionizing radiation and UV induction of p53 protein by different pathways in ataxia-telangiectasia cells. *Oncogene*, **8**, 3307-3312.

KUERBITZ SJ, PLUNKETT BS, WALSH WV AND KASTAN MB. (1992). Wild-type p53 is a cell cycle checkpoint determinant following irradiation. *Proc. Natl Acad. Sci. USA*, **89**, 7491-7495.

LEE JM AND BERNSTEIN A. (1993). p53 mutations increase resistance to ionizing radiation. *Proc. Natl Acad. Sci. USA*, **90**, 5742-5746.

LEVINE AJ, PERRY ME, CHANG A, SILVER A, DITTMER D, WU M AND WELSH D. (1994). The 1993 Walter Hubert Lecture: the role of the p53 tumour-suppressor gene in tumorigenesis. *Br. J. Cancer*, **69**, 409-416.

LI FP AND FRAUMENI JR JF. (1969). Soft-tissue sarcomas, breast cancer, and other neoplasms: a familial syndrome? *Ann. Intern. Med.*, **71**, 747-752.

LITTLE JB, NOVE J, DAHLBERG WK, TROILO P, NICHOLS WW AND STRONG LC. (1987). Normal cytotoxic response of skin fibroblasts from patients with Li-Fraumeni familial cancer syndrome to DNA-damaging agents *in vitro*. *Cancer Res.*, **47**, 4229-4234.

LOWE SW, SCHMITT EM, SMITH SW, OSBORNE BA AND JACKS T. (1993). p53 is required for radiation-induced apoptosis in mouse thymocytes. *Nature*, **362**, 847-849.

MAGNUSON NS, BECK T, VAHIDI H, HAHN H, SMOLA U AND RAPP UR. (1994). The Raf-1 serine threonine protein kinase. *Semin. Cancer Biol.*, **5**, 247-253.

MAKINO F AND OKADA S. (1975). Effects of ionizing radiations on DNA replication in cultured mammalian cells. *Radiat. Res.*, **62**, 37-51.

MALKIN D, LI FP, STRONG LC, FRAUMENI JR JF, NELSON CE, KIM DH, KASSEL J, GRYKA MA, BISCHOFF FZ, TAINSKY MA AND FRIEND SH. (1990). Germ line p53 mutations in a familial syndrome of breast cancer, sarcomas, and other neoplasms. *Science*, **250**, 1233-1238.

MARCU KB, BOSSONE SA AND PATEL AJ. (1992). *myc* function and regulation. *Annu. Rev. Biochem.*, **61**, 809-860.

MIRZAYANS R, WATERS R AND PATERSON MC. (1988). Induction and repair of DNA strand breaks and 1-β-D-arabinofuranosyl-cytosine-detectable sites in 40-75 kVp X-irradiated compared to $^{60}$Co γ-irradiated human cell lines. *Radiat. Res.*, **114**, 168-185.

MURNANE JP AND KAPP LN. (1993). A critical look at the association of human genetic syndromes with sensitivity to ionizing radiation. *Semin. Cancer Biol.*, **4**, 93-104.

PAINTER RB AND YOUNG BR. (1980). Radiosensitivity in ataxia-telangiectasia: a new explanation. *Proc. Natl Acad. Sci. USA*, **77**, 7315-7317.

PALCIC B AND JAGGI B. (1990). Image cytometry system for morphometric measurements of live cells. In *Bioinstrumentation: Research, Developments and Applications*, Wise DL (ed.) pp. 923-991. Butterworth: Stoneham, MA.

PARSHAD R, PRICE FM, PIROLLO KF, CHANG EH AND SANFORD KK. (1993). Cytogenetic response to $G_2$-phase X irradiation in relation to DNA repair and radiosensitivity in a cancer-prone family with Li-Fraumeni syndrome. *Radiat. Res.*, **136**, 236-240.

PATERSON MC, SMITH BP, LOHMAN PHM, ANDERSON AK AND FISHMAN L. (1976). Defective excision repair of γ-ray-damaged DNA in human (ataxia telangiectasia) fibroblasts. *Nature*, **260**, 444-447.

PATERSON MC, SELL BM, SMITH BP AND BECH-HANSEN NT. (1982). Impaired colony-forming ability following γ-irradiation of skin fibroblasts from tuberous sclerosis patients. *Radiat. Res.*, **90**, 260-270.

PATERSON MC, BECH-HANSEN NT, BLATTNER WA AND FRAUMENI JR JF. (1983). Survey of human hereditary and familial disorders for γ-ray response *in vitro*: occurrence of both cellular radiosensitivity and radioresistance in cancer-prone families. In *Radioprotectors and Anticarcinogens*, Nygaard OF and Simic MG (eds) pp. 615-638. Academic Press: New York.

PATERSON MC, GENTNER NE, MIDDLESTADT MV AND WEINFELD M. (1984). Cancer predisposition, carcinogen hypersensitivity, and aberrant DNA metabolism. *J. Cell. Physiol.*, **3** (Suppl.), 45-62.

PATERSON MC, MIDDLESTADT MV, WEINFELD M, MIRZAYANS R AND GENTNER NE. (1986). Human cancer-prone disorders, abnormal carcinogen response and defective DNA metabolism. In *Radiation Carcinogenesis and DNA Alterations* (NATO ASI Series A: Life Sciences, Vol. 124), Burns FJ, Upton AC and Silini G (eds) pp. 471-498. Plenum Press: New York.

PIROLLO KF, GARNER R, YUAN SY, LI L, BLATTNER WA AND CHANG EH. (1989). *Raf* involvement in the simultaneous genetic transfer of the radioresistant and transforming phenotypes. *Int. J. Radiat. Biol.*, **55**, 783-796.

PROKOCIMER M AND ROTTER V. (1994). Structure and function of p53 in normal cells and their aberrations in cancer cells: projection on the hematologic cell lineages. *Blood*, **84**, 2391-2411.

REED JC. (1994). Bcl-2 and the regulation of programmed cell death. *J. Cell Biol.*, **124**, 1-6.

SANGER F, NICKLEN S AND COULSON AR. (1977). DNA sequencing with chain-terminating inhibitors. *Proc. Natl Acad. Sci. USA*, **74**, 5463-5467.

SCHNEIDER EL, STANBRIDGE EJ AND EPSTEIN CJ. (1974). Incorporation of $^3$H-uridine and $^3$H-uracil into RNA: a simple technique for the detection of mycoplasma contamination of cultured cells. *Exp. Cell Res.*, **84**, 311-318.

SLICHENMYER WJ, NELSON WG, SLEBOS RJ AND KASTAN MB. (1993). Loss of a *p53*-associated G1 checkpoint does not decrease cell survival following DNA damage. *Cancer Res.*, **53**, 4164-4168.

SOUSSI T, DE FROMENTEL CC AND MAY P. (1990). Structural aspects of the p53 protein in relation to gene evolution. *Oncogene*, **5**, 945-952.

SOUSSI T, LEGROS Y, LUBIN R, ORY K AND SCHLICHTHOLZ B. (1994). Multifactorial analysis of *p53* alteration in human cancer: a review. *Int. J. Cancer*, **57**, 1-9.

SPENCER CA AND GROUDINE M. (1991). Control of c-*myc* regulation in normal and neoplastic cells. *Adv. Cancer Res.*, **56**, 1-48.

SRIVASTAVA S, ZOU Z, PIROLLO K, BLATTNER W AND CHANG EH. (1990). Germ-line transmission of a mutated *p53* gene in a cancer-prone family with Li-Fraumeni syndrome. *Nature*, **348**, 747-749.

SRIVASTAVA S, WANG S, TONG YA, HAO Z-M AND CHANG EH. (1993). Dominant negative effect of a germ-line mutant *p53*: a step fostering tumorigenesis. *Cancer Res.*, **53**, 4452-4455.

STEWART BW. (1994). Mechanisms of apoptosis: integration of genetic, biochemical, and cellular indicators. *J. Natl Cancer Inst.*, **86**, 1286-1296.

STRAUSS WM. (1994). Preparation of genomic DNA from mammalian tissue. In *Current Protocols in Molecular Biology*, Albright LM, Coen DM and Varki A (eds) pp. 2.2.1-2.2.3. John Wiley: New York.

TARONE RE, SCUDIERO DA AND ROBBINS JH. (1983). Statistical methods for in vitro cell survival assays. *Mutat. Res.*, **111**, 79-96.

YOUNG BR AND PAINTER RB. (1989). Radioresistant DNA synthesis and human genetic diseases. *Hum. Genet.*, **82**, 113-117.

WARENIUS HM, BROWNING PGW, BRITTEN RA, PEACOCK JA AND RAPP UR. (1994). C-*raf*-1 proto-oncogene expression relates to radiosensitivity rather than radioresistance. *Eur. J. Cancer*, **30A**, 369-375.